# The effects of melatonin on colonization of neonate spermatogonial mouse stem cells in a three-dimensional soft agar culture system

**DOI:** 10.1186/s13287-017-0687-y

**Published:** 2017-10-17

**Authors:** Shadan Navid, Mehdi Abbasi, Yumi Hoshino

**Affiliations:** 1 Department of Anatomy, School of Medicine, Tehran University of Medical Sciences, Tehran, Iran; 2 Laboratory of Reproductive Endocrinology, Graduate School of Biosphere Science, Hiroshima University, Higashi-Hiroshima, Kagamiyama 1-4-4, Hiroshima 739-8528 Japan

**Keywords:** Spermatogonial stem cell, Melatonin, Colonization, Three-dimensional soft agar culture system, Proliferation

## Abstract

**Background:**

Melatonin is a pleiotropic hormone with powerful antioxidant activity both in vivo and in vitro. The present study aimed to investigate the effects of melatonin on the proliferation efficiency of neonatal mouse spermatogonial stem cells (SSCs) using a three-dimensional soft agar culture system (SACS) which has the capacity to induce development of SSCs similar to in vivo conditions.

**Methods:**

SSCs were isolated from testes of neonate mice and their purities were assessed by flow cytometry using PLZF antibody. Isolated testicular cells were cultured in the upper layer of the SACS in αMEM medium in the absence or presence of melatonin extract for 4 weeks.

**Results:**

The identity of colonies was confirmed by alkaline phosphatase staining and immunocytochemistry using PLZF and α6 integrin antibodies. The number and diameter of colonies of SSCs in the upper layer were evaluated at days 14 and 28 of culture. The number and diameter of colonies of SSCs were significantly higher in the melatonin group compared with the control group. The levels of expression of ID-4 and Plzf, unlike c-kit, were significantly higher in the melatonin group than in the control group.

**Conclusions:**

Results of the present study show that supplementation of the culture medium (SACS) with 100 μM melatonin significantly decreased reactive oxygen species (ROS) production in the treated group compared with the control group, and increased SSC proliferation.

## Background

Spermatogonial stem cells (SSCs) are germline precursor cells with the potential to self-renew and generate differentiated germ cells which have the capability of producing progeny cells that form sperms [1]. A paucity of these stem cells in the mature testis has limited their isolation for in vitro studies [2]. In addition, long-term chemotherapy or radiation therapy in cancer patients has a devastating impact on these cells and can result in male infertility after the treatment. Therefore, methods for efficient in vitro proliferation of SSCs could play a critical role in sperm preservation and management of infertility, particularly among cancer survivors [3, 4]. Recent investigations have focused on the factors associated with the proliferation of cultured SSCs, such as leukemia inhibitory factor (LIF) [5, 6], glia cell line-derived neurotrophic factor (GDNF) [5, 7], basic fibroblast growth factor [5, 8], and stem cell factor [9, 10]. All of these factors have been found to play crucial roles in the development of SSCs in vitro. However, most of these experiments commonly investigated two-dimensional cell culture systems using culture dishes or flasks [11–13]. The main disadvantage of the two-dimensional model is the lack of metabolic and proliferative gradients similar to the natural SSC niche. In a three-dimensional culture system, Sertoli cells proliferate and create a monolayer of cells which forms a feeder layer on top of which SSCs colonies are formed [14]. It has been shown that three-dimensional culture systems improve cell proliferation [15]. Some studies have also demonstrated the importance of a three-dimensional structure on the proliferation and differentiation of animal and human SSCs [15–17]. The soft agar culture system (SACS) is a qualitative, three-dimensional cell culture structure that was first used for clonal expansion of bone marrow cells and exploration of factors that are associated with the regulation of their proliferation and differentiation [18, 19]. In 2008, Stukenborg et al. published a paper in which they used SACS as a novel approach to study factors involved in the regulation of the proliferation and differentiation of SSCs. This culture system provides a microenvironment similar to in vivo conditions and mimics some aspects of the natural three-dimensional environment to which the SSCs are exposed [15]. Melatonin (N-acetyl-5-methoxytryptamine) is considered as an important hormone with antioxidant, immune response, cell signaling, and neuroprotective properties [20, 21]. A normal physiological metabolism of cells generates reactive oxygen species (ROS), especially in the mitochondria, which can have adverse effects on cell signaling involved in the regulation of proliferation and survival [22]. Melatonin plays a critical role in reducing ROS production in cells, and inhibits a potential DNA mutation resulting from oxidative damage [23]. Similarly, Cruz et al. found that the amphiphilic nature of melatonin has an important implication in the protection of mammalian gametes and embryos from free radical-mediated oxidative damage and cellular death in vitro [24]. Melatonin receptors (MT1, MT2) are G protein coupled receptors. Several reports have indicated the expression of melatonin receptors in rat, mouse, sheep, bovine, and human Sertoli cells [25–28]. In this study, we obtained SSCs from the neonate mouse using two-step enzymatic digestion. The goal of the present study was to investigate the effects of melatonin (antioxidant) supplementation to the SACS culture medium containing LIF and GDNF on the proliferation of neonate mouse SSCs. Our findings show that SACS, along with the novel innovative medium used in this study, inhibits the release of free radicals during in vitro culture of SSCs and provides a promising strategy for the expansion of SSCs; the survival rate of SSCs was enhanced during a 4-week culture period using melatonin as an important antioxidant in the culture medium.

## Method

### Animals

Three- to 6-day-old NMRI (National Medical Research Institute) male mice were maintained under standard conditions. Animal experiments in this study were approved by the ethics committee of Tehran University of Medical Sciences in accordance with the university’s guidelines.

### Isolation of neonatal testis cells

Testicular cells were obtained from 3- to 6-day-old NMRI male mice. Testes were removed from the scrotum and tunica albuginea was removed to obtain the testis tissue. The testis tissues were cut into small pieces and transferred to the digestion solution containing collagenase type IV (1 mg/ml; Sigma, Germany), DNase (10 μg/ml; Sigma, Germany), and hyaluronidase (0.5 mg/ml; Sigma, Germany) for 20 min at 37 °C in a 5% CO_2_ incubator. Cells were dispersed by pipetting every 2–5 min until the tubules were separated. The dispersed cells were centrifuged at 1500 g for 5 min and then washed with phosphate-buffered saline (PBS). The second step of enzymatic digestion was carried out using the same procedure and enzymes (15 min). The isolated cells were washed again with PBS [29].

### Cell viability

The viability of testicular cells was assessed by methylthiazoltetrazolium (MTT; Sigma, Germany) before and after SSC culture in the SACS. Initially, the soft upper layer was removed and the cell pellet at the bottom of the 15-ml falcon tube was obtained by pipetting several times with PBS (heated to 37 °C). Following each pipetting, centrifugation was performed at 1500 g for 5 min. The isolated cells were counted using a hemocytometer. Finally, the cells were transferred into a 96-well culture plate with each well containing a density of 20 × 10^3^ cells per well per 200 μl; 400 μl MEM and 40 μl MTT were added to each well and incubated for 4 h at 37 °C in a 5% CO_2_ incubator. Following the incubation, the medium was replaced with 400 μl dimethyl sulfoxide (DMSO; 1.4 M; Sigma, Germany), and the cells were kept for 30 min at room temperature. The optical density (OD) of the plate was measured at 540 nm with the microplate reader.

### Intracellular ROS measurement

The general oxidative stress indicator CM-H2DCFDA was used for the detection of intracellular ROS production. DCFDA (10 ml; Sigma, Life Technologies C6827) was added to the cells and then incubated at 37 °C for 25 min. The DCFDA fluorescent probe reacts with intracellular H_2_O_2_ to generate fluorescence emission that can be detected by flow cytometry. Measurement of intracellular H_2_O_2_ production in SACS, before and after culture, was carried out by flow cytometry using DCFDA. The cells were washed twice with PBS and then centrifuged at 2500 g for 5 min. Green fluorescence emission was measured between 500 and 530 nm using flow cytometry [30].

### Flow cytometry

The efficiency and purity of the isolated SSCs were quantitatively assessed by flow cytometry. The two-step enzymatic digestion protocol yielded a single-cell suspension from 3- to 6-day-old male mice. The majority of these cells were SSCs. The cell suspension (density of 2 × 10^5^ cells/cm^2^) was transferred to a petri dish coated with gelatin and maintained for 24 h at 37 °C in a 5% CO_2_ incubator. The coated dish was then washed twice with PBS and centrifuged at 1500 g for 5 min. To assess the cellular enrichment percentage after the placement of the gelatin-coated dish, flow cytometry was performed by applying standard procedures and using a PLZF (promyelocytic leukemia zinc finger protein) antibody. Following permeabilization with 0.4% Triton X100 (Sigma), 10 μl of primary antibody (anti-PLZF antibody; Abcam, rabbit polyclonal to plzf) was added to the cells for 1 h at room temperature. The dish was again washed twice with 1 ml PBS, and 10 μl of secondary antibody (donkey anti-rabbit; Abcam) conjugated with fluorescein isothiocyanate (FITC) was added (1 h at 4 °C). In the control cells, the primary antibody was omitted [31].

### SACS

The SSC culture was prepared according to the procedure used by Stukenborg et al. [15]. The SACS was composed of two layers: the soft layer (upper) and solid layer (lower). The single-cell suspension was added to the soft upper layer (0.37% (w/v) agar) established on the solid lower layer (0.5% (w/v) agar), and cultured in a 24-well plate. The cells (10^6^ cells per well per 200 μl) were cultured for 4 weeks in the upper layer of the soft agar medium consisting of 0.37% agar plus the basic culture medium αMEM containing 10% fetal bovine serum (FBS; Sigma, Germany), 1× nonessential amino acids (Invitrogen, USA), 0.1 mM 2-mercaptoethanol (Sigma, Germany), 100 U/ml penicillin (Sigma, Germany), 100 μg/ml streptomycin (Sigma, Germany), 10^3^ U/ml human recombinant leukemia inhibitory factor (LIF; B&D, USA), and 10 μg/ml glial cell line-derived neurotrophic factor (GDNF; R&D, USA). The final volume of the upper layer was 200 μl. The solid lower layer had 0.5% (w/v) agar plus the basic culture medium αMEM containing 10% fetal bovine serum only. Prior to culturing, the solid layer was formed and kept for 2 h at 37 °C in a 5% CO_2_ incubator. The final volume of the lower layer was 800 μl. Cell suspension was added to the αMEM culture medium before mixing with the agar. The agar and the αMEM culture medium were mixed at 37 °C and the cells settled on top of the solid lower layer. In the treatment group, melatonin at 50 μM or 100 μM (Sigma) was added to the basic medium culture [32]. All culture experiments were maintained in standard cell culture incubators at 37 °C and 5% CO_2_. At the end of days 14 and 28 (the second and fourth week after seeding in the two groups), the diameter and number of colonies formed were measured. We separately counted the colonies in each well of the same group (4–6 wells in each group). The culture medium was replaced every 3 days. Three techniques were used to confirm the presence of colonies of SSCs: alkaline phosphatase staining, immunocytochemistry using plzf and α6 integrin antibodies, and the level of expression of the undifferentiated genes ID-4 and Plzf using real-time polymerase chain reaction (PCR).

### Alkaline phosphatase staining

Alkaline phosphatase activity was assayed by Fast Red TR/Naphthol AS-MX Tablets (Sigma USA, F4648) according to the manufacturer’s instructions. Alkaline dye was briefly made by dissolving a Tris tablet in 1 ml of deionized water to form a Tris buffer, after which a Fast-Red TR/Naphthol AS-MX Tablet was added. The cells were added to the alkaline dye and incubated at 37 °C for 30 min. Subsequently, the cells were observed under an inverted microscope.

### Immunocytochemistry for characterization of SSC colonies

Immunocytochemical detection of the PLZF antibody was used to identify the colonies formed by the SSCs. After fixation with 4% paraformaldehyde for 24 h, the cells were permeabilized with 0.4% Triton X100 (Sigma) and then blocked in 10% goat serum (Sigma). The cells were then incubated with two primary antibodies, rabbit polyclonal anti-PLZF (1:100; Abcam) and rat polyclonal anti-α6-integrin (1:100; Sigma-Aldrich), for 2 h at 37 °C. After 2 h of incubation, the cells were washed with PBS and the secondary antibody, donkey anti-rabbit or anti-rat labeled with FITC and diluted at 1:200 (Sigma), was added for 3 h. Control cells were treated under similar conditions except for the removal of the primary antibodies. Nuclei were stained with 4,6-diamidino-2-phenylindole (DAPI; 1 μg/ml; Sigma, Germany).

### Real-time PCR

After 4 weeks of culture, the expression levels of promyelocytic leukaemia zinc finger protein (*Plzf*, undifferentiated gene), DNA-binding protein inhibitor (*ID4*, undifferentiated gene), and tyrosine-protein kinase Kit (*c*-*kit*, differentiated gene) were measured by real-time PCR. Total RNA was extracted by Trizol reagent (Ready Mini Kit, Qiagen, USA) according to the manufacturer’s instructions. Total RNA (1 μg) was applied for cDNA synthesis using a cDNA synthesis kit (Transcript First Strand cDNA Synt, Roche, USA) according to the manufacturer’s guidelines. Real-time PCR was carried out in 40 reaction amplification cycles, and Applied Bioscience 7500 fast with SYBR Green detection was used for the analysis. Melt curve analysis was performed after each run to detect the presence of nonspecific PCR products and primer dimers. All samples were normalized against glyceraldehyde-3-phosphate dehydrogenase (GAPDH) (internal control), and the relative quantification of gene expression was determined using the comparative CT method (ΔΔCT). Primer sequences for RT-PCR are listed in Table 1.

**Table 1:** The primer sequences for ID4, Plzf, c-kit, and GAPDH genes

Gene name	Sequence	Product size (bp)	Annealing temperature (° C)
*ID4*	Forward: 5'- TCCCGCCCAACAAGAAAGTC -3'	102	60.54
	Reverse: 5'- TCAGCAAAGCAGGGTGAGTC-3'		
*Plzf*	Forward: 5'- CGTTGGGGGTCAGCTAGAAAG -3'	301	57.14
	Reverse: 5'- CACCATGATGACCACATCGC-3'		
*c*-*kit*	Forward: 5'- AACAACAAAGAGCAAATCCAGG -3'	200	57.67
	Reverse: 5'- GGAAGTTGCGTCGGGTCTAT -3'		
*GAPDH*	Forward: 5'-AGCAAGGACACTGAGCAAGAG-3'	151	60.35
	Reverse: 5'- TCGTTCCTCTGATCGTTTCC -3'		

### Statistical analysis

Results are expressed as the mean ± standard deviation (SD). Statistical analysis was performed using the unpaired *t* test for the gene expression studies. The comparison of the diameter and number of colonies, cell viability, and ROS measurements between the test groups were performed by repeated analysis of variance (ANOVA) followed by a Tukey post-hoc test for internal comparisons. *P* ≤ 0.05 was considered statistically significant.

## Results

### Assessment of the purification of SSCs by flow cytometry

**Fig. 1:**
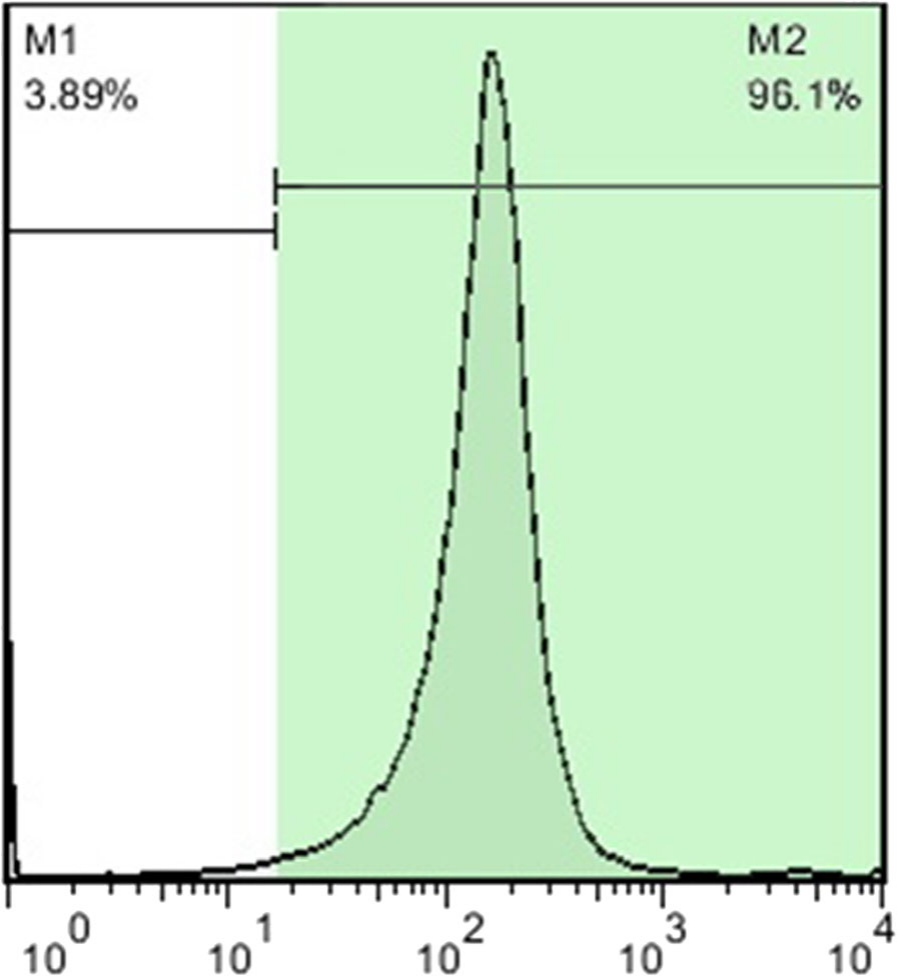
Flow cytometry analysis for the detection of the percentage of purity of SSCs with Plzf marker after the placement of the dish coated with gelatin. M1: PLZF-negative cells, M2: PLZF-positive cells

A flow cytometry technique was carried out on the supernatant after the placement of the gelatin-coated dish to assess the percentage of purity of SSCs following the two-step enzymatic digestion. We observed that 96.1% of all cells expressed Plzf (Fig. 1) which is one of the most suitable markers for the isolation of undifferentiated SSCs [33]. Other cells of the testes, predominantly Sertoli cells, that were transferred into the SACS played important roles in the formation of colonies of SSCs.

### Effect of SACS on the viability of SSCs

**Fig. 2:**
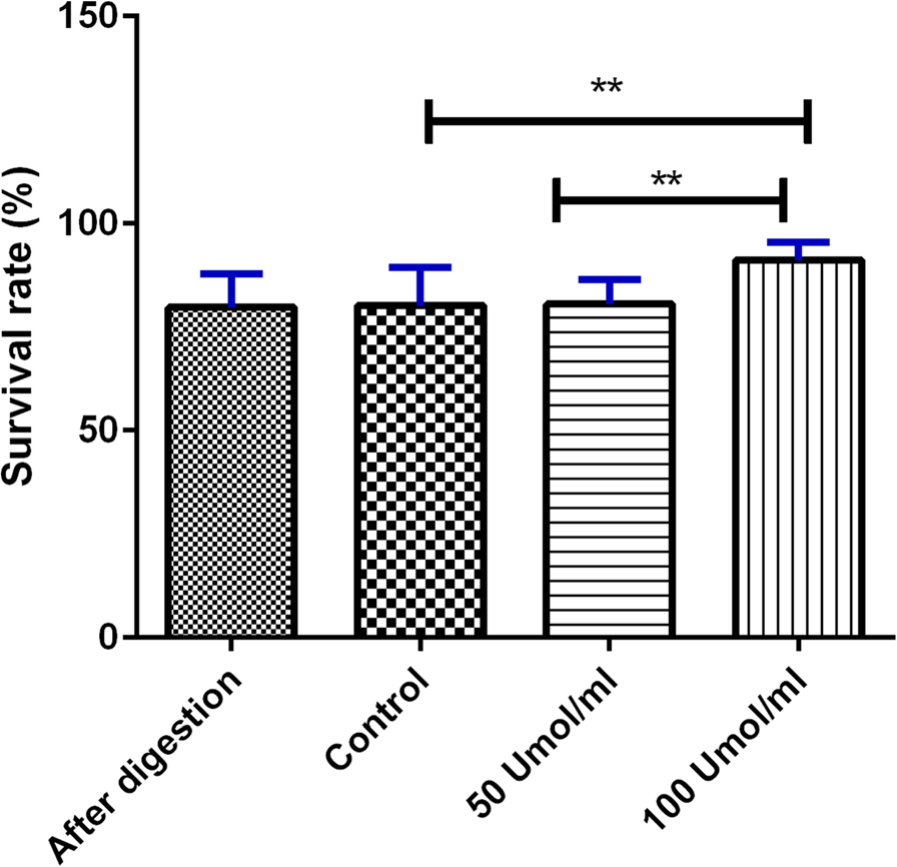
MTT analysis for assessment of SSC viability in different treatment groups. Data are expressed as means ± SD. **P ≤ 0.01

The MTT assay was used to evaluate the viability of cells. The results show that the majority of the cells (91.32 ± 4.2%) were viable immediately after the two-step enzymatic digestion (*P* ≤ 0.05). After 4 weeks of culture of SSCs in the SACS, the survival rates of the cells in the control group (80.13 ± 9%) and 50 μM melatonin group (80.57 ± 5.8%) were not significantly different compared with the fresh cells group (79.8 ± 8%; *P* ≥ 0.05). However, cell viability was significantly (*P* ≤ 0.05) increased when 100 μM melatonin (91.11 ± 4.3%) was added to the culture medium compared with the control (80.13 ± 9%) group. This concentration was thus used for further studies (Fig. 2).

### Evaluation of SSC development using the SACS

**Fig. 3:**
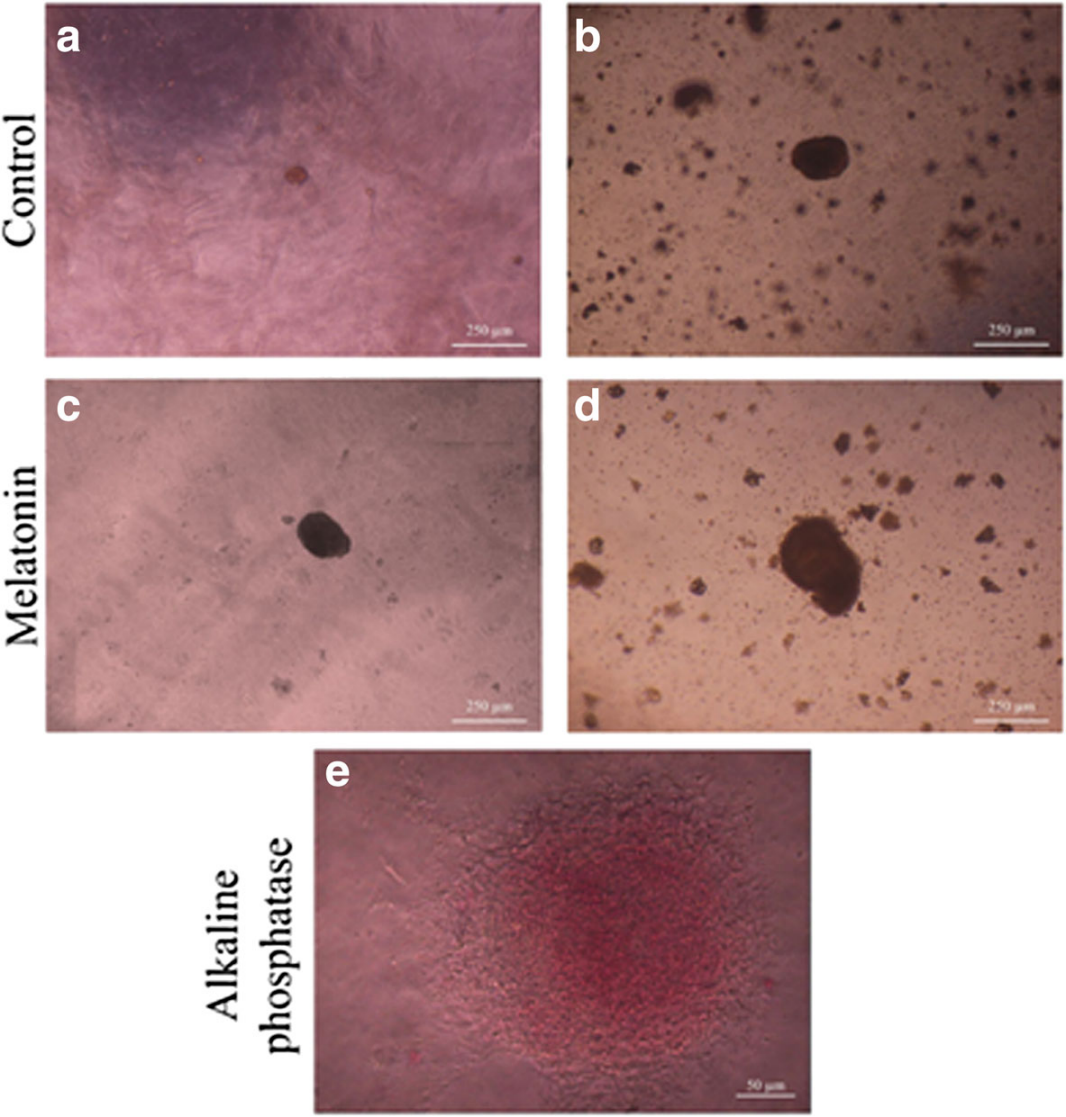
Microscopic morphology of SSCs derived from neonatal male mice (five mice at a time in each group). Each type of experiment has three replicates. The sizes of the colonies are shown at the end of the second week (**a**, **c**) and the end of the fourth week (**b**, **d**) of SACS, as well as being positive for alkaline phosphatase activity (**e**). After cultivation of SSCs, the diameter and number of colonies increased in both groups, particularly in the experimental (melatonin) group. All the tests performed at least five times

**Fig. 4:**
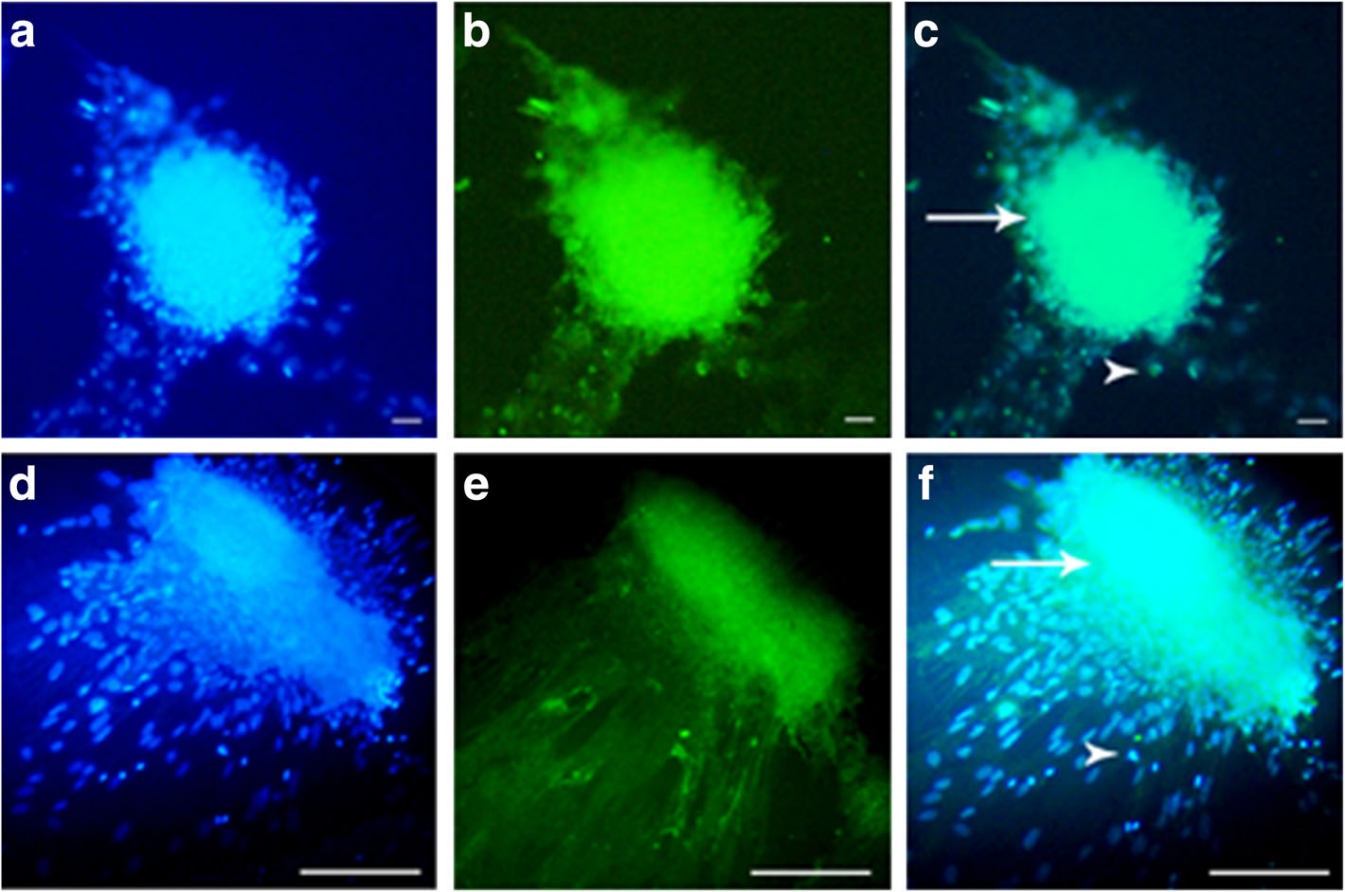
Immunofluorescent staining of SSC colonies. SSCs were positive for Plzf in the nuclei and α6 integrin in the cytoplasm (*green*) (**b**, **e**), and nuclei were stained with DAPI (*blue*) (**a**, **d**). **c**, **f** A merge of Plzf and α6 integrin DAPI. A single SSC cell (*arrowhead*) proliferated and created a colony of SSCs (*arrow*). *Scale bars* = 50 μm

**Fig. 5:**
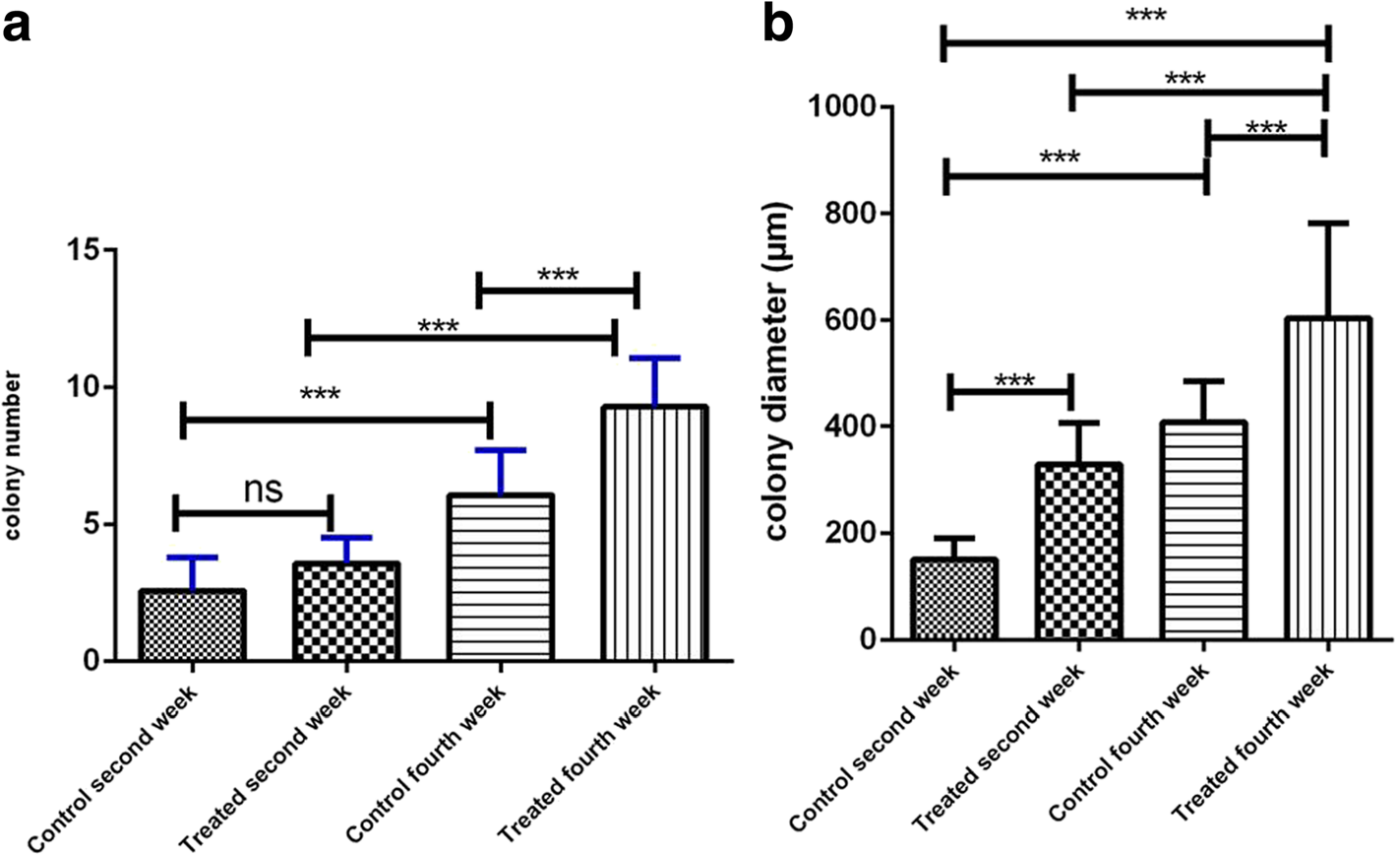
Comparison of colony numbers (**a**) and diameter (**b**) between the control and antioxidant groups. Data are expressed as means ± SD. ***P ≤ 0.001

After the two-step enzymatic digestion, we cultured the testicular cells for 4 weeks. Isolated testicular cells were cultured in the upper layer of the SACS. Distinct colonies appeared in the upper layer of the SACS between days 9 and 11 during the culture period. The diameter and number of colonies in the upper layer were measured at the end of second and fourth week (days 14 and 28 after seeding in the two groups) (Fig. 3a–d). SSC colonies appeared between days 9 and 11 in the experimental group, and on day 13 in the control group. Alkaline phosphatase activity of the isolated colonies strongly suggests a well-accepted embryonic characterization of the stem cells (Fig. 3e). Moreover, for the identification of colonies of SSCs in the SACS we detected the expression levels of PLZF and α6 integrin by immunocytochemistry, and this was confirmed by the presence of PLZF protein in the nuclei and α6 integrin protein in SSC cytoplasm, respectively (Fig. 4). The number and diameter of colonies in the upper layer were compared between the groups on days 14 and 28 of culture. In the colony assay, the number and diameter of colonies in the control group were 2.57 ± 1.2 and 150.7 ± 39.5 μm, respectively, at the end of the second week, and 6.07 ± 1.6 and 407.7 ± 77.3 μm, respectively, at the end of the fourth week. In the experimental group, the number and diameter of colonies were 3.57 ± 0.9 and 329.9 ± 76.5 μm, respectively, at the end of the second week, and 9.28 ± 1.7 and 602.8 ± 179.5 μm, respectively, at the end of the fourth week. The number and diameter of colonies formed in the experimental group after the fourth week of culture were significantly higher than in the control group (*P* ≤ 0.001), although the differences were not significant at the end of the second week. These results show that melatonin supplementation in the culture medium, along with SACS, can mimic in vivo conditions and has a significant effect on the diameter and number of SSC colonies (Fig. 5).

### Gene expression analysis

**Fig. 6:**
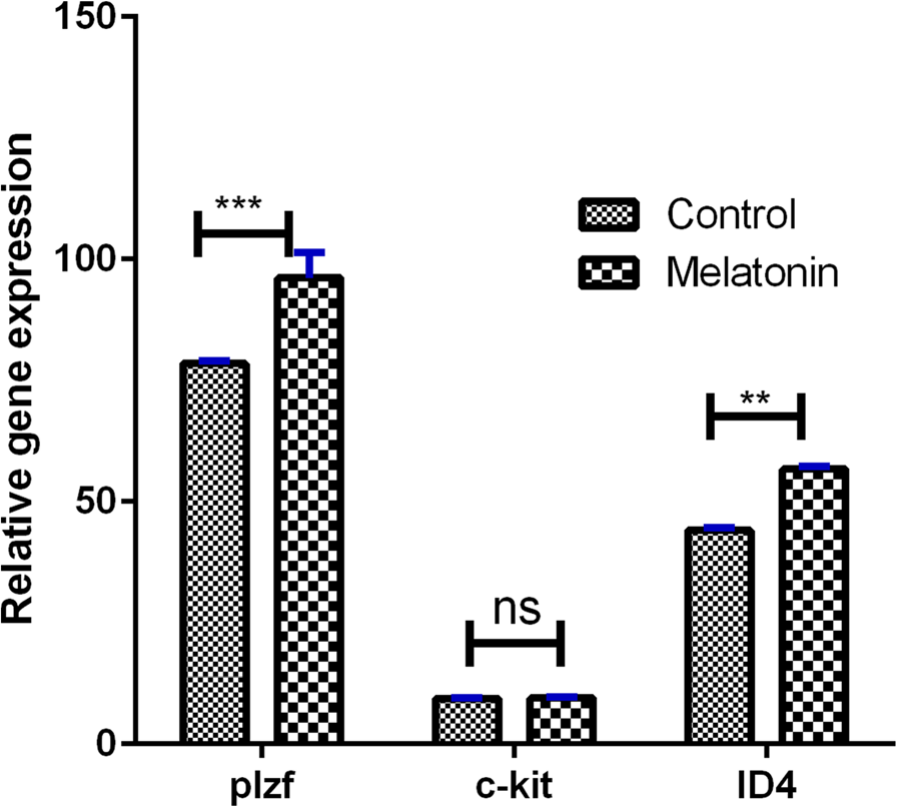
Expression pattern of *c*-*kit*, *Plzf*, and *ID4* genes after culture (with antioxidant) analyzed by real-time PCR. Levels of *ID4* and *Plzf* in the antioxidant group had an increased value compared with the control group, but the level of *c*-*kit* in the antioxidant group had no significant differences compared with the control group. Data are expressed as means ± SD. **P ≤ 0.01, ***p ≤ 0.001. *ns* not significant

Expression levels of Plzf, ID-4, and c-kit were examined using real-time PCR for the evaluation of the proliferation of SSCs in the SACS. The results indicate that the levels of Plzf and ID-4 expression in the melatonin group were significantly higher than in the control group (Plzf, *P* ≤ 0.001 vs. control; ID-4, *P* ≤ 0.01 vs. control). C-kit gene had a lower level of expression in the melatonin group compared with the control group; however, the difference was not significant (*P* ≥ 0.05) (Fig. 6).

### Flow cytometric evaluation of intracellular ROS production

**Fig. 7:**
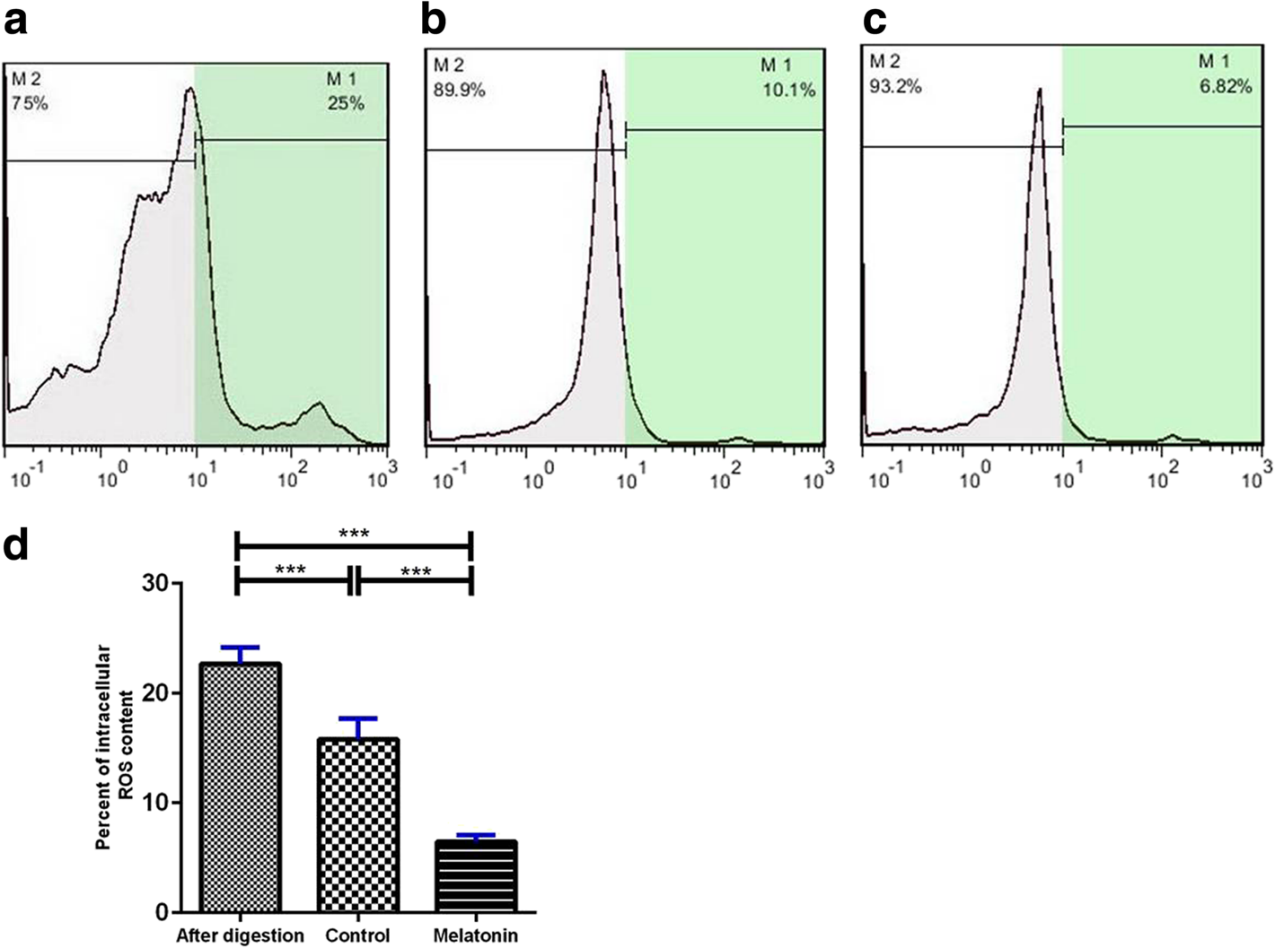
Flow cytometry analysis for the detection of reactive oxygen species (*ROS*) in different groups. **a** Fresh group, **b** control group (without melatonin), **c** experimental group (melatonin 100 μM). M2: DCFDA-negative cells, M1: DCFDA-positive cells. **d** ROS production of SSCs before and after SACS analyzed by flow cytometry. Note the significantly lower production of ROS after SACS with 100 μM melatonin compared to the control group. Data are expressed as means ± SD. ***P ≤ 0.0001

To measure intracellular ROS production, we used DCFDA which is a specific probe for intracellular H_2_O_2_ detection. Measurement of intracellular ROS production before and after SACS was carried out by flow cytometry using the DCFDA probe. ROS production in the fresh group (25.7 ± 0.8%) was significantly (*P* ≤ 0.0001) higher compared with the control (10.67 ± 0.7%) and melatonin groups (6.46 ± 0.5%) (Fig. 7).

## Discussion

The present study makes noteworthy contributions by providing valuable tools for the in vitro investigation of SSC proliferation which can be useful in the treatment of male infertility. In the present study, we developed SSC culture in SACS along with melatonin supplementation as the optimal culture protocol which prevented the release of free radicals during spermatogonial stem cell culture in vitro. In a previous study, the number and diameter of colonies increased in a group treated with melatonin in SACS compared with a two-dimensional culture supplemented with date palm pollen (*Phoenix dactylifera*), which confirms our study describing a successive maturation of pre-meiotic SSCs in the culture system. Neonatal mouse SSCs were isolated and enriched with Plzf antibody, which was also used to confirm SSC colonies in the SACS. Plzf antibody has been used in many studies as an indicator for SSC colony detection and purification studies [14, 34]. Several other studies have used different markers for the isolation and detection of these cells, including GFRα1, ID-4, PAX7, etc. [35–37]. However, there is no strong evidence for the efficient isolation of SSCs using these markers, and no efficient and specific SSC markers have yet been identified [38].

The SACS consisted of two phases of different agar concentrations: a softer upper layer and a more solid lower layer. The synthesis of SACS was performed according to the procedure applied by Stukenborg et al. [15].

In this study, we added SSCs to the upper layer of the agar system. Similarly, Elhija et al. [17], Stukenborg et al. [15], and Huleihel et al. [39] reported the addition of SSCs (10^6^ cells per well per 200 μl) to the upper layer of the agar system before culturing in a 24-well plate during their investigations on SSC proliferation. Unlike these studies, we only used a flow cytometry technique for the assessment of the purity of SSCs using Plzf antibody; 96.1% of all cells expressed this antibody. Other cells of the testes, especially Sertoli cells, were transferred into the upper layer of the SACS which shows that these cells have positive effects on development and colony formation in SSCs [15]. Sertoli cells are involved in the regulation of proliferation and differentiation of SSCs, particularly through paracrine- and endocrine-mediated signaling pathways. Sertoli cell growth factor, GDNF, fibroblast growth factor 2 (FGF2), Sertoli cell transcription factor, ETS variant 5, nociceptin, neuregulin 1 (NRG1), and androgen receptor (AR) have been identified as the most important upstream factors that regulate SSC self-renewal and spermatocyte meiosis [40]. Recent studies have demonstrated the expression of melatonin receptors (MT1 and MT2) in Sertoli cells [25] and SSCs [28]. Thus, the fact that melatonin, in addition to its antioxidant activity, can have complex biological functions through its receptor appears to be justified. Based on these findings, it seems likely that melatonin, through its receptor on Sertoli cells or SSCs, can directly play a role in the proliferation of SSCs. The isolated cells were cultured in SACS in the absence or presence of melatonin extract. Their viability was evaluated by MTT assay. In this study, we observed a dose-dependent (100 μM) activity of melatonin on SSCs in culture. As previously published, the presence of melatonin in a cell culture can increase the number of viable cells [20, 28]. We added melatonin to the basic culture medium in the SACS, which yielded an increase in cell viability up to 90%. Our results also show that the number of viable cells in the SACS supplemented with melatonin was higher compared with a two-dimensional culture system supplemented with catalase or alpha-tocopherol before culture [41]. We also examined the effects of SACS in combination with LIF and GDNF on the proliferation of SSCs. Although several reports have described culturing SSCs in SACS, they have most commonly focused on stem cell differentiation [15, 17, 39]. Previous studies have shown that some growth factors, most notably GDNF, can have a long-term positive effect on the maintenance of SSCs and may also stimulate division of SSCs [7, 12, 42, 43]. Other studies reported that LIF is an essential factor for maintaining pluripotency and the self-renewing capacity of embryonic stem cells [44] and SSCs [45]. On the other hand, the presence of LIF in the culture medium inhibited meiotic gene expression and increased the percentage of alkaline phosphatase-positive cells. Previous studies have also demonstrated that melatonin can play different roles in the cells of the body such as cell signaling, protection of fatty acids from oxidation, oncostatic action, and antiapoptotic and anti-aging properties in many cells [21, 46]. In recent years, much attention has focused on the role of melatonin as an antioxidant. Melatonin, as a free radical scavenger, plays a vital role in the reduction of ROS production, and prevents cellular death and potential DNA mutations resulting from oxidative damage in culture systems [20, 23, 26, 47–49]. One of the most common intracellular ROS molecules is H_2_O_2_ [50]. We thus evaluated intracellular H_2_O_2_ content using a DCFDA-specific probe. Our findings demonstrate that supplementation with melatonin in SACS can contribute greatly to the prevention of the propagation of lipid peroxidation in SSC membranes caused by ROS production, and can protect SSCs from the adverse effects of these free radicals. Conversely, Morimoto et al. [51] reported that ROS formation plays a pivotal role in SSC self-renewal via the activation of stress kinases p38 mitogen-activated protein kinase (MAPK) and c-Jun N-terminal kinase (JNK) pathways. They suggested that the presence of ROS is somewhat necessary for SSC self-renewal in vivo [51].

In another study, Li et al. [52] claimed that melatonin promotes manganese superoxide dismutase (*MnSOD*) and sirtuin type 1 (*SIRT1*) expression, and therefore promotes busulfan-induced SSC apoptosis in the presence of high concentrations of ROS and p53.

A number of studies have demonstrated that the addition of melatonin to culture medium can reduce the potential effects of ROS-induced cell stress in cells such as oocyte and adipose-derived stem cells (ASCs) [48, 49]. Gholami et al. [53] carried out a number of investigations on the effects of melatonin on SSC transplantation in azoospermic mice. They showed that melatonin can improve the structure of testis tissue. In another study conducted by the same researchers [54], they demonstrated positive effects of melatonin supplementation in vitrified-thawed testicular germ cells of neonatal mice. They indicated that melatonin can induce cell proliferation in normal cells and apoptosis in damaged cells. Similarly, Niu et al. [55] found that adding melatonin to the culture medium of goat SSCs could increase SSC proliferation by stimulating the production of GDNF in the Sertoli cell. Measuring the number and diameter of colonies of SSCs can be used as morphological criteria in in vitro studies [41, 56]. In another study, we investigated the effect of melatonin on SSCs in a two-dimensional culture, and emphasized on the importance of adding melatonin to the culture medium as an antioxidant. We also showed that the culture of SSCs in SACS is more successful compared to the two-dimensional culture system [57].

In this study, we observed that the number and diameter of the colonies increased at the end of each week of culture in the SACS in both groups. The most striking aspect of our results is the vital antioxidant role of melatonin in the culture of SSCs in the SACS which provided protection against lipid peroxidation. Similar to our results, Elhija et al. [17] isolated colonies in the upper layer of SACS after 14 and 28 days of culture and classified them according to their size. In contrast to our findings, Eslahi et al. [16] indicated that the number and diameter of the colonies of SSCs in a poly-l-lactic acid (PLLA) nanofiber scaffold decreased significantly after the first, second, and third weeks of culture compared with the control groups (culture of SSCs not seeded on PLLA). In the present study, colonies were composed of cells that expressed and stained positive for the mitosis markers PLZF and α6 integrin. Moreover, we used alkaline phosphatase staining to evaluate the colonies of SSCs for alkaline phosphatase activity. It is well known that PLZF and α6 integrin are markers for spermatogonial stem/progenitor cells in many species [14, 16, 45].

After the fourth week of culture, we analyzed ID-4, Plzf, and c-kit gene expression levels. Our data clearly show that melatonin supplementation can increase the proliferation rate of SSCs. The level of PLZF and ID-4 (undifferentiated genes) expression in the melatonin group were significantly higher than in the control group, whereas the level of c-kit (differentiated gene) expression decreased in the melatonin group. In support of our results, Aliakbari et al. also reported a decrease in c-kit expression after they cultured post-thawed SSCs treated with antioxidants in both control and treated groups [41]. Our findings are in line with the results of other previous studies [14, 16, 58].

Results of this research suggest several practical applications. We emphasize the importance of adding melatonin to the culture medium as an antioxidant.

## Conclusions

We conclude that the presence of LIF and GDNF in SACS, along with melatonin supplementation in the basic culture medium, likely creates a testis-like microenvironment in which proliferation of SSCs is promoted. On the other hand, the major limitation of this study was the inability to isolate live cells from the SACS. Therefore, we could not investigate later stages, and further study is needed to evaluate their differential fertility potential which is the gold standard for the management of those suffering from infertility.

## Abbreviations

c-kit: Tyrosine-protein kinase Kit
FBS: Fetal bovine serum
FITC: Fluorescein isothiocyanate
GDNF: Glia cell line-derived neurotrophic factor
ID-4: DNA-binding protein inhibitor
LIF: Leukemia inhibitory factor
MTT: Methylthiazoltetrazolium
NMRI: National Medical Research Institute
PBS: Phosphate-buffered saline
PCR: Polymerase chain reaction
PLZF: Promyelocytic leukemia zinc finger protein
ROS: Reactive oxygen species
SACS: Soft agar culture system
SSC: Spermatogonial stem cell
